# Preserved Electroencephalogram Power and Global Synchronization Predict Better Neurological Outcome in Sudden Cardiac Arrest Survivors

**DOI:** 10.3389/fphys.2022.866844

**Published:** 2022-04-20

**Authors:** Li-Ting Ho, Bess Ma. Fabinal Serafico, Ching-En Hsu, Zhao-Wei Chen, Tse-Yu Lin, Chen Lin, Lian-Yu Lin, Men-Tzung Lo, Kuo-Liong Chien

**Affiliations:** ^1^ Division of Cardiology, Department of Internal Medicine, National Taiwan University College of Medicine and Hospital, Taipei, Taiwan; ^2^ Institute of Epidemiology and Preventive Medicine, College of Public Health, National Taiwan University, Taipei, Taiwan; ^3^ Department of Biomedical Sciences and Engineering, National Central University, Taoyuan, Taiwan

**Keywords:** electroencephalography, neurological outcome, prognosis, sudden cardiac arrest, phase coherence, global synchronization

## Abstract

Quantitative EEG (qEEG) delineates complex brain activities. Global field synchronization (GFS) is one multichannel EEG analysis that measures global functional connectivity through quantification of synchronization between signals. We hypothesized that preservation of global functional connectivity of brain activity might be a surrogate marker for good outcome in sudden cardiac arrest (SCA) survivors. In addition, we examined the relation of phase coherence and GFS in a mathematical approach. We retrospectively collected EEG data of SCA survivors in one academic medical center. We included 75 comatose patients who were resuscitated following in-hospital or out-of-hospital nontraumatic cardiac arrest between 2013 and 2017 in the intensive care unit (ICU) of National Taiwan University Hospital (NTUH). Twelve patients (16%) were defined as good outcome (GO) (CPC 1–2). The mean age in the GO group was low (51.6 ± 15.7 vs. 68.1 ± 12.9, *p* < 0.001). We analyzed standard EEG power, computed EEG GFS, and assessed the cerebral performance category (CPC) score 3 months after discharge. The alpha band showed the highest discrimination ability (area under curve [AUC] = 0.78) to predict GO using power. The alpha band of GFS showed the highest AUC value (0.8) to predict GO in GFS. Furthermore, by combining EEG power + GFS, the alpha band showed the best prediction value (AUC 0.86) in predicting GO. The sensitivity of EEG power + GFS was 73%, specificity was 93%, PPV was 0.67%, and NPV was 0.94%. In conclusion, by combining GFS and EEG power analysis, the neurological outcome of the nontraumatic cardiac arrest survivor can be well-predicted. Furthermore, we proved from a mathematical point of view that although both amplitude and phase contribute to obtaining GFS, the interference in phase variation drastically changes the possibility of generating a good GFS score.

## 1 Introduction

The electroencephalogram (EEG) is a commonly available, reasonably inexpensive, and noninvasive diagnostic technique that may be used to assess cognitive impairment. Although EEG recordings do not reveal particular abnormalities in the most prevalent causes of cognitive impairment, they may be useful when a diagnosis is in dispute. Currently, monitoring EEG activity frequently or continuously for SCA (sudden cardiac arrest) survivors has been suggested as part of a general guideline in neurological prognostication, which provides information that helps clinicians provide the best treatments based on the patient’s likelihood of gaining a neurologically meaningful recovery (Nolan et al., 2021). SCA indicates the abrupt loss of heart function, breathing, and consciousness, usually resulting from the heart’s electrical system. Despite advances in the treatment of heart diseases, the outcome of SCA survivors remains poor (Chan et al., 2014; Daya et al., 2015; Writing Group Memebers, 2016). Hypoxic–ischemic encephalopathy (HIE) is caused by systemic hypoxemia and/or reduced cerebral blood flow (CBF). It most often results from cardiac arrest, vascular catastrophe, poisoning, or head trauma in adults and birth asphyxia in the neonates. Survivors of SCA may have variable degrees of hypoxic–ischemic brain injury, depending on the duration of circulatory arrest, the extent of resuscitation efforts, and underlying comorbidities. The EEG activity of SCA survivors may show characteristic patterns, depending on the severity of the hypoxic–ischemic brain injury, which may play a part in the prediction of the neurological outcome. The EEG waveform is a complex rhythmic activity requiring specific expertise. Some patterns are very clear and easy to be interpreted by clinicians, such as suppression, burst suppression, periodic/rhythmic discharge, and evolving seizure activity. However, some patterns, such as discontinuous background and unreactive background, may be misclassified by a less-experienced physician. The computational processing of EEG, also called quantitative EEG (qEEG), has demonstrated its power in delineating the complex EEG waveform resulting from the brain activities. The EEG data analysis provides novel and effective techniques for comprehending the dynamic complexity of the EEG time series. These qEEG analyses extract the signal in frequency-, space-, and time-domain, where information about the signal can be used to assess as a biological measure. These parameters can be utilized for better neurological outcome prediction after cardiac arrest.

Furthermore, a proposed measurement uses multichannel EEG to get a measure of global phase alignment as a function of frequency across all derivations. The interaction comes in the form of a particular level of synchronization. Synchronization of multivariate systems refers to the adjustment of one given property between these systems to reach a common behavior between them due to the coupling process (Cimenser et al., 2011; Achermann et al., 2016). Global field synchronization (GFS) is a scale that assesses phase synchronization across all derivations and goes from 0 (no predominant phase; least phase synchronization across derivations) to 1 (maximum phase synchronization among derivations; perfect phase synchronization). Given that the EEG signal is not generated by a single, focal electric source, the presence of a predominant phase across scalp measurements implies that the intracranial neuroelectric dynamics have a preferred phase as well and that a phase spread across scalp measurements was caused by intracranial electric sources that differed in phase (Koenig et al., 2001). Koenig et al. (2005) and Ma et al. (2014) looked into the values of GFS in Alzheimer’s disease (AD) patients compared to healthy individuals. In AD, as the disease progresses, connections between brain networks break down as a result of the shrinking of many brain regions. GFS, in particular, deals with the synchronization between these brain networks. The significant decline of the GFS value in patients with AD compared to healthy individuals holds considerable potential of serving as an indicator of cognitive impairment in patients with AD. Furthermore, Smailovic et al. (2018) investigated the correlation of GFS and other qEEG measures with conventional cerebrospinal fluid (CSF) biomarkers associated with AD. GFS in some frequency bands, particularly fast frequency bands, exhibited significant correlation with CFS. It decreased in AD as compared to the control group. Their findings provided evidence that these GFS measures can be potential markers of AD.

However, the explanation of how and why phase synchronization affects the level of GFS was not discussed thoroughly in these past studies. In general, perfect phase synchronization is damaged when the oscillators are in the presence of noise, which is unavoidable in experimental or real systems. These fluctuations differ with different channels that affect the GFS strength. By investigating these interferences, a deeper understanding of how GFS works can be evaluated as well as understanding special features that are not observed in the synchronization of periodic oscillation. In addition, previous studies calculated the GFS of the whole signal. We proposed segmentation of the signal for better filtration of noise, which lessens the effect of noise on GFS results.

In this study, we planned to perform a retrospective cohort study to examine whether qEEG and GFS may serve as a reliable predictor for outcomes after cardiac arrest. We hypothesized that loss of global functional connectivity of brain activity might be a surrogate marker for poor outcome in SCA survivors. In addition, phase synchronization and GFS relation was investigated in a mathematical approach. Moreover, we looked into the implementation of new methods in improving GFS strength in signals with noise interference.

## 2 Methods and Materials

### 2.1 Subjects and Study Protocol

All patients with nontraumatic cardiac arrest after cardiopulmonary and cerebral resuscitation (CPCR) were screened for study inclusion. The patients were treated by standardized post-resuscitation treatment based on clinical conditions. Based on the post-resuscitation protocol in our institute, the feasibility of target temperature management (TTM) of all sudden cardiac death patients should be evaluated. If the post-resuscitation GCS is less than M5, TTM should be applied, unless the patient has contraindications of TTM such as very unstable hemodynamic status or severe bleeding tendency. Due to facility limitation, these patients received a standard EEG examination in an EEG room. According to the post-resuscitation protocol, standard EEG would be arranged on day 3 and day 7 after resuscitation, including patients who received TTM. If the patient’s consciousness fully recovered after resuscitation, the EEG will be canceled. For patients with clinical suspicions of nonconvulsive seizures or status epilepticus by the neurologist, repeated EEG will be arranged to increase the yield rate. EEG signals with a signal-to-noise ratio (SNR) computed as the power within the lowest frequency and the highest frequency bands of interest (0.15–35 Hz) over the total power of the rest of the signal less than 12 dB are considered having poor EEG quality and were excluded from the study. The neurological outcome was assessed at discharge and 3 months after discharge based on clinical records. Survivors with a cerebral performance category (CPC) score of 1–2 were classified as “good outcome”, and patients with a CPC score of 3–5 or who died during the hospitalization were classified as “poor outcome” (Perkins et al., 2015; Geocadin et al., 2019).

We included 75 comatose patients who were resuscitated following in-hospital or out-of-hospital nontraumatic cardiac arrest between 2013 and 2017 in the intensive care unit (ICU) of National Taiwan University Hospital (NTUH). The mean EEG performing day was day 4 after cardiac arrest. All patients who had good EEG quality were included for further analysis. A total of 12 patients (16%) were defined as good outcome. A total of 32 patients were defined as poor neurological outcome, and 31 patients died in the hospital stay. Most of the patients who underwent the EEG exam were sedation-free. Only 11 patients had sedatives at the time of the EEG exam. Within these 11 patients, two had good neurological outcome, five had poor neurological outcome, and four patients died. Patients with poor neurological outcome and mortality (63 patients, 84%) were defined as poor outcome.

### 2.2 Mathematical Model of Global Field Synchronization

In this section, we established the mathematical relationship between GFS and phase coherence. It was to prove that GFS is mainly determined by the phase difference between signals. In addition, the influence of noise in GFS was explained and demonstrated. Finally, we proposed an anti-noise algorithm to solve the problem concerning noise.

We provided a brief description of band-limited signals and the covariance matrix from complex Fourier transform of multiple channels in the succeeding sections.

#### 2.2.1 Covariance Matrix Approximation in Calculation of Global Field Synchronization

An arbitrary band-limited signal x(t), which includes an amplitude modulation and a frequency modulation term, is represented as

$$x\left(t\right)=A\left(t\right)e^{j\left(\mathrm{\omega t}+\varphi \left(t\right)\right)}.$$ (1)

The signal x(t)  can be filtered and divided into different frequency distributions around a center frequency ω restricted by frequency modulation φ(t) . Moreover, the amplitude modulation A(t) could be time-varied or stably distributed.

Global field synchronization (GFS) is a method developed to observe the functional connectivity (FC) between different EEG channels. It locates the synchronization at a specific frequency band by applying the general signal model to multichannel signals.

GFS can be calculated as follows: The multichannel α -band EEG signals’ frequency response is represented as

$${ℱ}_{a}\left\{x_{i_{\alpha }}\left(t\right)\right\}=a_{i}e^{j\theta_{i}},$$ (2)

where $\;x_{i_{\alpha }}\left(t\right)=A_{i}\left(t\right)e^{j\left(\omega_{\alpha }t+\varphi_{i}\left(t\right)\right)}$ and the phase $\theta_{i}$ is a random variable of uniform distribution from −η∼η .

Fourier transform was then applied to the signal. The Fourier transform ℱ of x(t) at specific frequency ${\text{ω}}_{a}$ is defined as

$${ℱ}_{a}\left\{x\left(t\right)\right\}\;=\int A\left(t\right)e^{j\left({\text{ω}}_{\alpha }t+\varphi \left(t\right)\right)}e^{-j{\text{ω}}_{\alpha }t}dt=\int A\left(t\right)e^{j\varphi_{i}\left(t\right)}dt=ae^{j\theta }.$$ (3)

The signal then yields a complex form made up of real and imaginary parts for each frequency given as

$$\begin{array}{c} Re\left\{{ℱ}_{a}\left\{x_{i_{\alpha }}\left(t\right)\right\}\right\}=a_{i}\cos\left(\theta_{i}\right)\\ \mathrm{Imag}\left\{{ℱ}_{a}\left\{x_{i_{\alpha }}\left(t\right)\right\}\right\}=a_{i}\sin\left(\theta_{i}\right) \end{array}.$$ (4)

For every particular frequency band, the pair of resulting complex Fourier coefficients from all channels could be mapped as points in a two-dimensional diagram. To calculate the degree of diversion on the real-complex diagram, principal component analysis (PCA), a best-fit straight-line approximation, was used.

Matrix $D_{\alpha }$ , comprising the real and imaginary part of the frequency response of N channels, is represented as

$$D_{\alpha }=\left[\begin{array}{c} D_{\alpha -1}\\ D_{\alpha -i}\\ \begin{array}{c} \vdots \\ D_{\alpha -K} \end{array} \end{array}\right]\;\;\;s.t.\;\;\;\;D_{\alpha -i}=a_{i}\cdot {\left[\begin{array}{c} \cos\left(\theta_{i}\right)\\ \sin\left(\theta_{i}\right) \end{array}\right]}^{T}\;\;\;\;\forall \;K\in z\;\;\exists \;\;0\leq \;i\;\leq K.$$ (5)

Then, principal components can be derived from the covariance matrix of $D_{\alpha }$ matrix given as

$$\sum \left(D_{\alpha }\right)=D_{\alpha }^{T}D_{\alpha }=\left[\begin{array}{cc} \sum a_{i}^{2}cos^{2}\left(\theta_{i}\right) & \sum a_{i}^{2}sin\left(\theta_{i}\right)cos\left(\theta_{i}\right)\\ \sum a_{i}^{2}sin\left(\theta_{i}\right)cos\left(\theta_{i}\right) & \sum a_{i}^{2}sin^{2}\left(\theta_{i}\right) \end{array}\right].$$ (6)

Finally, the GFS score can be calculated using the ratio of eigenvalue on the real-complex diagram.

$$\lambda_{\alpha }=\mathrm{eigenvalue}\left(D_{\alpha }^{T}D_{\alpha }\right),$$ (7)

$$GFS_{\alpha }=\frac{\left|\lambda_{\alpha 1}-\lambda_{\alpha 2}\right|}{\lambda_{\alpha 1}+\lambda_{\alpha 2}},\;\;\;\;\;\;\;\;\mathrm{where}\;\lambda_{\alpha 1}>\lambda_{\alpha 2}.$$ (8)

The GFS score reveals whether the components on the real-complex diagram can be concluded with single or two components. The more dominant the first component of the real-complex diagram, the more synchronized the different brain areas.

Furthermore, the relationship of GFS with extended number of channels can also be defined. It is assumed that there are K channel signals. The calculation of the real-complex matrix D (Eq. 6) and its covariance matrix Σ is the same as in Eq. 7, and it is assumed that the phase distribution η  between channels is small.

if η<δ, where δ≅0≥0 

$$\theta_{i}≅0,\;\sin\left(\theta_{i}\right)≅0.$$

Σ could be a defective matrix (Eq. 9), which has only one eigenvalue. Therefore, the distribution on the real-complex diagram yields to the single phase and higher GFS score, while signals of different channels have strong connectivity.

$$\Sigma ≅\left[\begin{array}{cc} 1 & Q\\ Q & 0 \end{array}\right]\in Defective\;Matrix\;,\;Q\in R\;\;\;s.t.\;\;Single\;Eigenvector\;\&\;\;GFS\;↑.$$ (9)

Moreover, we provided mathematical proof for GFS derivation with the zero-mean process before performing principal component analysis.

$D_{\alpha }$ Matrix Would Become $D_{z}$

$$D_{z}=D_{\alpha }-\mu \;s.t\;\;D_{z-i}=\left[\begin{array}{cc} a_{i}\cos\left(\theta_{i}\right)-\mu_{x} & a_{i}\sin\left(\theta_{i}\right) \end{array}-\mu_{y}\right],$$ (10)

where $\mu_{x}$ represents the average magnitude of the real part in every channel and $\mu_{x}$ represents the average magnitude of the imaginary part.

$$\mu_{x}=\frac{1}{N}\sum a_{i}\cos\left(\theta_{i}\right)\;\;\;\;\;\;\;\;\;\;\;\;\;\mu_{y}=\frac{1}{N}\sum a_{i}\sin\left(\theta_{i}\right),$$ (11)

$$\sum \left(D_{z}\right)=\left[\begin{array}{cc} var\left(Re\{{ℱ}_{a}\{x_{i_{\alpha }}\left(t\right)\}\right\}) & cov\left(Re\{{ℱ}_{a}\{x_{i_{\alpha }}\left(t\right)\}\right\},Im\left\{{ℱ}_{a}\left\{x_{i_{\alpha }}\left(t\right)\right\}\right\})\\ cov\left(Re\{{ℱ}_{a}\{x_{i_{\alpha }}\left(t\right)\}\right\},Im\left\{{ℱ}_{a}\left\{x_{i_{\alpha }}\left(t\right)\right\}\right\}) & var\left(Im\{{ℱ}_{a}\{x_{i_{\alpha }}\left(t\right)\}\right\}) \end{array}\right].$$ (12)

Assuming that the phase distribution η  between channels is small, $\mu_{x}$ will be approximated as the mean amplitude of each channel, while the $\mu_{y}$ will equal to zero.

$$\begin{array}{c} \mathrm{If \eta}<\mathrm{\delta ,}\;\mathrm{where}\;\delta ≅0\geq 0\;\\ \theta_{i}≅0,\;\sin\left(\theta_{i}\right)≅0\\ \mu_{x}=\frac{1}{N}\sum a_{i}\cos\left(\theta_{i}\right)\approx \bar{a}\;\;\mu_{y}=\frac{1}{N}\sum a_{i}\sin\left(\theta_{i}\right)\approx 0 \end{array}.$$ (13)

Eventually, the covariance matrix, following the previous result, will obtain a high GFS score and eigenvector with only narrow phase distribution.

$$\begin{array}{c} \sum \left(D_{z}\right)=\left[\begin{array}{cc} \sum {\left(a_{i}-\bar{a}\right)}^{2} & \sum \left(a_{i}-\bar{a}\right)\left(a_{i}\sin\left(\theta_{i}\right)\right)\\ \sum \left(a_{i}-\bar{a}\right)\left(a_{i}\sin\left(\theta_{i}\right)\right) & 0 \end{array}\right]=\left[\begin{array}{cc} n\cdot var\left(a\right) & Q\\ Q & 0 \end{array}\right]\\ \in Defective\;Matrix,\;Q\in R\;\;\;\;\;s.t.\;\;Single\;Eigenvector\;\&\;\;GFS\;↑ \end{array}.$$ (14)

On the other hand, considering the uniform amplitude signals, the amplitude between channels has a narrow variation, so $a_{1}=a_{2}=a_{3}=\ldots .=a_{i}$ and var(a)≈0 .

$$\sum \left(D_{z}\right)\approx \left[\begin{array}{cc} 0 & 0\\ 0 & 0 \end{array}\right].$$ (15)

The covariance matrix of $D_{z}$ will turn out to be a zero matrix. Meanwhile, the principal component analysis becomes an ill-posed problem. GFS will fail to evaluate the degree of synchronization.

#### 2.2.2 Mathematical Analyzation of the Effect of Phase Coherence to Global Field Synchronization

GFS is defined by the phase synchronization among different channels across the brain. In this section, an in-depth evaluation on the relationship of phase coherence, a method to evaluate interaction between paired-signals, and GFS was discussed. In addition, the mechanism of synchronization was also analyzed.

A band-limited signal x(t) could be acquired by applying a band-pass filter to the signal and then Hilbert transform to access its phase.

Two arbitrary signals and their phases are defined as

$$x_{1_{\alpha }}\left(t\right)=A_{1}\left(t\right)e^{j\left(\omega_{\alpha }t+\varphi_{1}\left(t\right)\right)},$$ (16)

$$x_{2_{\alpha }}\left(t\right)=A_{2}\left(t\right)e^{j\left(\omega_{\alpha }t+\varphi_{2}\left(t\right)\right)},$$ (17)

where $\phi_{1}\left(t\right)=\omega_{\alpha }t+\varphi_{1}\left(t\right)\;$ and $\phi_{2}\left(t\right)=\omega_{\alpha }t+\varphi_{2}\left(t\right)$ .

Then, the phase coherence is formulized as

$$Coh_{1-2}=\frac{1}{N}\left|\sum e^{j\left(\phi_{1}-\phi_{2}\right)}\right|.$$ (18)

From the aforementioned signal model, the phase coherence assumes that when the paired signals start interacting, the modulation terms of the two signals, $\varphi_{1}\left(\text{t}\right)$ and $\varphi_{2}\left(\text{t}\right)$ , start off with a similar pattern. This produces a small phase difference resulting in increase in coherence. Therefore, the phase coherence is also used to determine the degree of synchronization.

Furthermore, the relationship between phase coherence and global field synchronization (GFS) was presented from a mathematical viewpoint. The phase coherence emphasizes on the similarity of frequency modulation function, while GFS adapts Fourier transform to approximate signal and focus on the phase, which is integrated from frequency modulation function by time.

For instance, it is assumed that each channel signal is phase-locking. The frequency modulation terms in different signals $\varphi_{i}\left(\text{t}\right)$ are equal and make the phase coherence high.

$$\begin{array}{c} \mathrm{∵Phase}\;\mathrm{Lockng}\;∴\;\varphi_{i}\left(t\right)=\varphi_{j}\left(t\right)=\varphi \left(t\right)\\ s\mathrm{.t}.\;\;\;Coh_{i-j}=\frac{1}{N}\left|\sum e^{j\left(\phi_{i}-\phi_{j}\right)}\right|\;↑ \end{array}.$$ (19)

Then, the Fourier transform of different signals at a given frequency can also be observed.

$$\begin{array}{c} {Χ}_{i}={ℱ}_{a}\left\{A_{i}\left(t\right)e^{j\left(\omega_{\alpha }t+\varphi_{i}\left(t\right)\right)}\right\}=A_{K}e^{j\theta_{i}}\\ ∵\;\varphi_{i}\left(t\right)=\varphi \left(t\right)\;\;∴\theta_{i}\approx \theta \;\\ s.t.\;\;A_{K}e^{j\theta_{i}}=A_{K}e^{j\theta }\;\;GFS\;↑\\ \mathrm{where}\;\theta \;\epsilon \;Random\;variable\;\;\left|\theta \right|<\mathrm{\eta ,\eta}≅0 \end{array}.$$ (20)

In this case, because the frequency modulation function $\varphi_{K}\left(\text{t}\right)$ is similar, $\theta_{K}$ could appear as a uniformly distributed sample with small variation ( η≅0 ), that is, the multichannel could also obtain an elongated distribution on a complex domain and share a higher GFS score.

On the contrary, if the signals in different channels are not in sync, the frequency modulation term $\varphi_{i}\left(\text{t}\right)$ would be in various patterns. Furthermore, the phase $\theta_{i}$ from Fourier transform would also have a more random distribution ( η>Q Q∈R ). Then, both phase coherence and GFS may reflect a poor score.

$$\begin{array}{c} ∵\mathrm{Random}\;\mathrm{modulation}\;s.t\;\phi_{i}\neq \phi_{j}\;\\ s.t.Coh_{i-j}=\frac{1}{N}\left|\sum e^{j\left(\phi_{i}-\phi_{j}\right)}\right|\;↓\\ {Χ}_{i}={ℱ}_{a}\left\{A_{i}\left(t\right)e^{j\left(\omega_{\alpha }t+\varphi_{i}\left(t\right)\right)}\right\}=A_{K}e^{j\theta_{i}}\;\;\;\\ \mathrm{where}\;\;\theta_{i}\;\epsilon \;Random\;variable\;\;\;\left|\theta_{i}\right|<\mathrm{\eta ,\eta}>Q\\ ∴GFS\;↓ \end{array}.$$ (21)

#### 2.2.3 Noise*-*Resistant Algorithm Application to Global Field Synchronization

Noise artifact is a critical issue in the domain of EEG signal processing. In this section, we provided a system to deal with the noise. Signal $x_{i}\;$ is represented by

$$x_{i}=a_{i}e^{i(\omega_{\alpha }t+\varphi_{i}\left(t\right))}+n\left(t\right).$$ (22)

The Fourier transform of $\;x_{i}$ is $ℱ\left\{x_{i}\right\}=a_{i}e^{j\theta_{i}}+Ce^{j{\text{Θ}}_{i}}$ . If all channel signals are phase coherent, then $\theta_{i}$ is a random variable within a small variation η . However, the Fourier transform of $x_{i}$ is sensitive to the background noise phase. Then, ${\text{Θ}}_{i}$ , a random variable generated from noise (i.e, n(t) ), will significantly affect the GFS score.

To prevent interference from white noise, we adapted the ensemble strategy in the frequency domain to enhance the SNR of GFS. We could divide k segmentations of each channel as different epochs to generate the GFS.

$$\begin{array}{c} s_{i}\left(t\right)=\overset{M}{\underset{k=1}{\sum }}s_{i,k}\left(t\right)\\ s_{i,k}=s_{i}\left(t\right)\times \Pi \left(\frac{t-kT}{T}\right),\;T\in window\;length \end{array}.$$ (23)

To reconstruct the whole signal’s GFS score from the segmented part, the segment GFS should be compensated with a time-delay factor, Ψ . Each GFS signal component $s_{i,k}\left(t\right)$ can be represented in the following form:

$$s_{i,k}\left(t\right)=F^{-1}\left\{F\left\{a_{i}e^{j\left(\omega_{\alpha }\left(t+KT\right)+\varphi_{i}\left(t\right)\right)}+n_{i,k}\left(t\right)\right\}\cdot \Psi \right\},\;\;\Psi =e^{-\text{j}\omega_{\alpha }\mathrm{KT}},$$ (24)

$$\;s_{i,k}\left(t\right)=a_{i}e^{j\left(\omega_{\alpha }\left(t+KT\right)+\varphi_{i}\left(t\right)\right)}+n_{i,k}\left(t\right)\cdot e^{-\text{j}\omega_{\alpha }\mathrm{KT}},$$ (25)

$$\approx a_{i}e^{j\left(\omega_{\alpha }\left(t\right)+\varphi_{i}\left(t\right)\right)}+\hat{n_{i,k}}\left(t\right)=x_{i,k}\left(t\right)+\hat{n_{i,k}}\left(t\right).$$ (26)

The ensemble frequency response at $\omega_{\alpha }$ is derived from the ensemble signal model. The GFS score could be recalculated from the ensemble frequency response.

$$ℱ\left\{s_{i}\left(t\right)\right\}=\frac{1}{M}\overset{M}{\underset{1}{\sum }}ℱ\left\{x_{i,k}\left(t\right)\right\}\;+\frac{1}{M}F\left\{\hat{n_{i,k}}\left(t\right)\right\}\;\;\mathrm{at}\;\;\omega_{\alpha }.$$ (27)

To demonstrate the advantage of the ensemble-average method, we need to discuss the performance in the signal part $x_{i,k}\left(t\right)$ and noise part $\hat{n_{i,k}}\left(t\right)$ independently.

First, we could read the signal part in a channel. The average of signal component frequency response could be written as

$$ℱ\left\{x_{i}\left(t\right)\right\}=\frac{1}{M}\overset{M}{\underset{1}{\sum }}ℱ\left\{x_{i,k}\left(t\right)\right\}\;at\;\omega_{\alpha }=a_{i}e^{j\xi_{i}},$$ (28)

$$ℱ\left\{x_{i,k}\left(t\right)\right\}=\int a_{i,k}e^{j\left(\omega_{\alpha }\left(t-KT\right)+\varphi_{i}\left(t\right)\right)}e^{-j\omega_{\alpha }t}dt=a_{i,k}e^{j\theta_{i,k}}.$$ (29)

The phase of each signal segment $\theta_{i,k}$ is a random variable with uniform distribution from –η∼η . As mentioned in Section 2.2.1, the value of η depends on the degree of synchronization between k segments. Signal $x_{i}\left(t\right)$ should have a wide sense stationary process condition to satisfy the operation of the ensemble average. Moreover, the phase modulation function $\varphi_{i}\left(t\right)$ in $x_{i}\left(t\right)$ observes the ergodic process, that is, the phase modulation function in different segments $\varphi_{i,k}\left(t\right)$ resembles each other and the modulation function $\varphi_{i}\left(t\right)$ of all signals. Eventually, the phase of the Fourier integral will stabilize.

$$\begin{array}{c} ∵\;\mu_{x}=E\left(x_{i}\left(t\right)\right)=E\left(x_{i,k}\left(t\right)\right)=\gamma \;,\;\gamma \in R\\ \&\;\;E\left(\varphi_{i}\left(t\right)\right)=E\left(\varphi_{i,k}\left(t\right)\right)\\ s.t.\;\;\;\;\theta_{i}=\theta_{i,k} \end{array}.$$

Thus, the ensemble average process would not jeopardize the phase performance.

The distribution of $\xi_{i}$ would be correlated to the phase distribution $\theta_{i,k}$ in different channels. $\;\xi_{i}$ is a random variable with uniform distribution from −η∼η .

$$\xi_{i}=f\left(\theta_{i}\right)=f\left(\theta_{i,k}\right).$$

With that, the incoherence phase (i.e., large probability distribution of $\;\theta_{i}$ ) among different channels remains chaotic after segmentation average. In other words, the low GFS caused by weak phase coherence should not be enhanced by ensemble average.

After discussing the signal part, we also consider noise contribution to GFS. The average of each noise segment frequency response could be written as

$$ℱ\left\{\hat{n_{i,k}}\left(t\right)\right\}=\frac{1}{M}\overset{M}{\underset{1}{\sum }}ℱ\left\{\hat{n_{i,k}}\left(t\right)\right\}\;at\;\omega_{\alpha }=\hat{C_{i}}e^{j\phi_{i}},\;\;C_{i}=\frac{C}{\sqrt{M}}.$$ (30)

Noticeably, the operation of ensemble average is able to attenuate the noise components. As long as the number of segmentations is enough, the noise influence to the GFS can be attenuated.

### 2.3 Electroencephalogram Quantification

We used standard EEG for analysis. The electrodes were attached at 19 standard sites on the scalp (international 10/20 system placements Fp1/2, F3/4, C3/4, P3/4, O1/2, F7/8, T3/4, T5/6, Fz, Cz, and Pz). We separated the EEG frequency bands into delta (0.5-4Hz), theta (4-8Hz), alpha (8-12Hz), and beta (12-40Hz). All EEGs were interpreted by neurologists, and the patterns were defined according to the ACNS EEG terminology (Westhall et al., 2016) as qualitative EEGs. Suppression, burst-suppression, periodic discharges, rhythmic discharges, and evolving seizure activity are highly malignant patterns. Discontinuous background, low-voltage, and unreactive background are malignant patterns. Continuous background indicates good outcome, which is defined as continuous normal voltage (>20 μV) background and preserved reactivity to stimuli.

For quantitative EEG analysis, for each frequency band, we averaged the value in the frequency domain and obtained the data around the middle of the recording in the time domain to avoid the artifact caused by emergency treatment. This value was taken as quantitative EEG. For each frequency band, 19 points of quantitative EEG were acquired.

We computed GFS by transforming an EEG signal with M channels into the frequency domain using Fourier transform (FT) (Eq. 3). Then, the FT of the signal yields a complex form made up of real (Eq. 4) and imaginary parts (Eq. 5) for each frequency. For every particular frequency band, pairs of resulting complex Fourier coefficients from all channels can be mapped as points in a two-dimensional diagram. The resulting pairs of all channels   were then quantified using principal component analysis (PCA) resulting in two eigenvalues per frequency. Using these eigenvalues (Eq. 8), we compute GFS as the ratio of the resulting eigenvalues (Eq. 9). For a more thorough step-by-step explanation, refer to Section 2.2.1.

### 2.4 Statistical Analysis

The data were expressed as mean ± standard deviation for continuous variables and percentage for categorical variables. The baseline characteristics between good vs poor outcome were compared by using *t*-test for continuous variables and chi-square test for categorical variables. Logistic regression analysis was performed to find the predictors for outcome. The baseline characteristics including age, gender, out-of-hospital cardiac arrest (OHCA), shockable rhythm, cardiopulmonary resuscitation (CPR) duration, use of target temperature management (TTM), use of extracorporeal membrane oxygenation (EMCO), Acute Physiology and Chronic Health Evaluation II (APACHE II) score, and comorbidities such as coronary artery disease (CAD), diabetes mellitus (DM), hypertension (HTN), chronic kidney disease (CKD), and atrial fibrillation (AF) were entered as covariates. Receiver operating characteristic (ROC) analysis was used to determine the optimal prediction model and evaluate the ability of GFS to discriminate good from poor outcome with the area under curve (AUC) as an indicator. Sample size analysis of the area under ROC curve using Medcalc Version 20.027, with type I error 0.05, type II error 0.2, area under ROC curve 0.8, and ratio of sample size in negative/positive groups 5, estimated the sample size was 54, in which nine were defined as good outcome and 45 were defined as poor outcome.

## 3 Results

### 3.1 Amplitude and Phase Interference to Global Field Synchronization

In the previous sections, we discussed the covariance matrix (Eq. 7) and approximated the signal model with narrow phase variation (Eq. 16) to prove that the synchronized phase has a single eigenvalue and high GFS score, that is, we could assume that phase coherence is the dominant factor to GFS.

#### 3.1.1 Numerical Global Field Synchronization Results From Two Dimensions

Aside from the approximated model proof (Eq. 16), we also performed numerical experiments to demonstrate the general performance of GFS. We inspected whether amplitude or phase is the critical interference to GFS. In this simulation, we acquired GFS values from the general form of the covariance matrix (Eq. 7). The amplitude of different channels, $a_{i},\;$ can be determined as a random variable of Gaussian distribution with different standard deviation ( σ: 0–5). The phase of different channels, $\theta_{i},$ can be set as a random variable of normal distribution with different range ( 0∼4 ).

**FIGURE 1:**
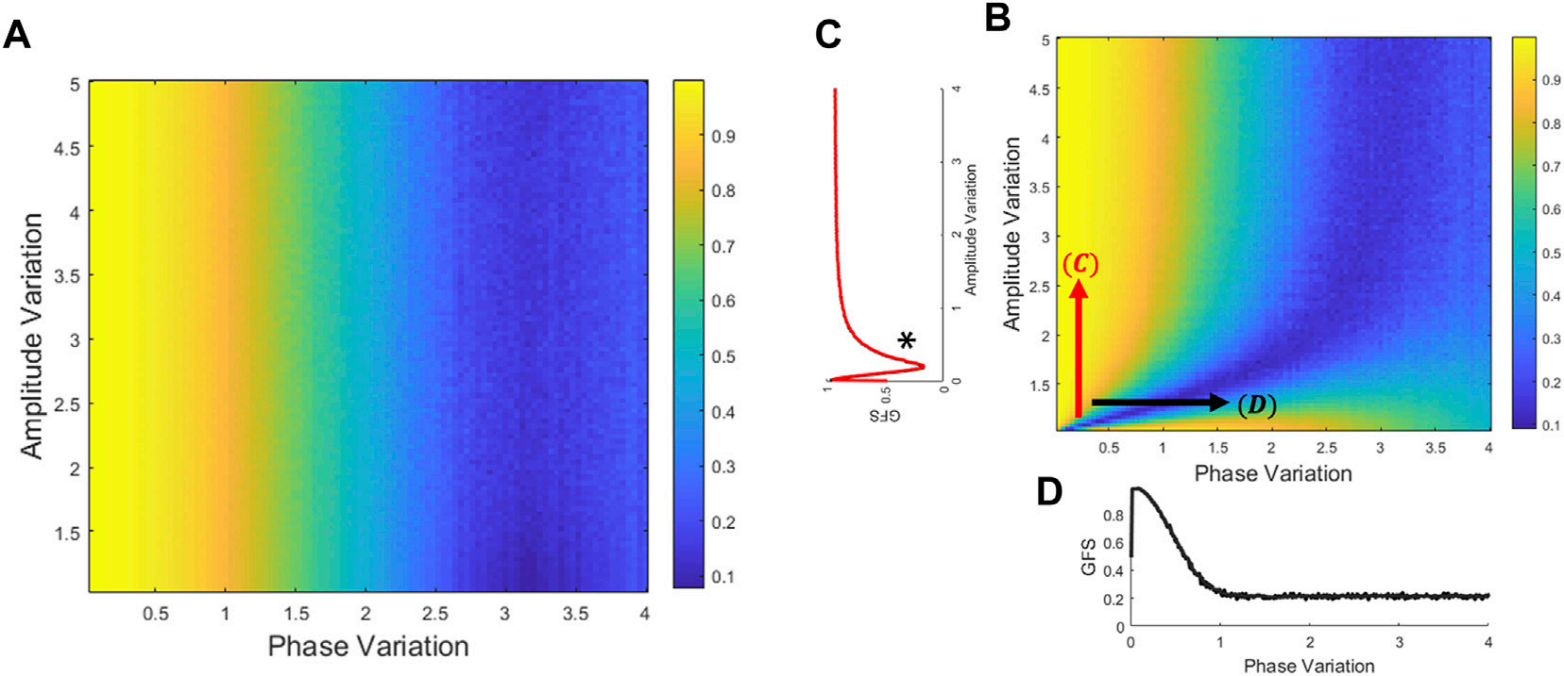
Relation of GFS to variations of phase and amplitude. The GFS score could be derived from the theoretical covariance matrix **(** Eqs. 7–13 **)**. Generate amplitude and phase distribution to compare the GFS performance between amplitude and phase perturbation. **(A)** GFS was derived from the covariance matrix without the zero-mean process **(** Eqs. 6–8 **)**. **(B)** GFS was derived from the covariance matrix with the zero-mean process **(** Eqs 10–13 **)**. **(C)** In the phase locking signals, it is on the left side of the * that the amplitude variation is dominant to the GFS. Therefore, the GFS fails to evaluate the phase synchronization with extremely small amplitude distribution. **(D)** In most of the amplitude distribution, GFS is sensitive to phase but not to the amplitude.

Numerical simulations from the theoretical proof demonstrate our assumptions for both GFS derived from the covariance matrix without zero-mean process (Figure 1A) and with zero-mean process (Figure 1B). Figure 1A shows that while the GFS score exhibits no significant difference with the increase in amplitude variation, increase in phase variation causes drastic decrease in the resulting score. Moreover, Figure 1B reveals that in extremely small amplitude distribution, the GFS fails to evaluate the phase synchronization. Taking everything into account, the GFS method without the zero-mean process overcomes the narrow amplitude limitation of GFS. In other words, if the signals have enough distribution of amplitude, the zero-mean process has no effect on GFS. Furthermore, although both derivations have small differences, phase variation is still a critical issue both to traditional GFS and GFS without the zero-mean process.

**FIGURE 2:**
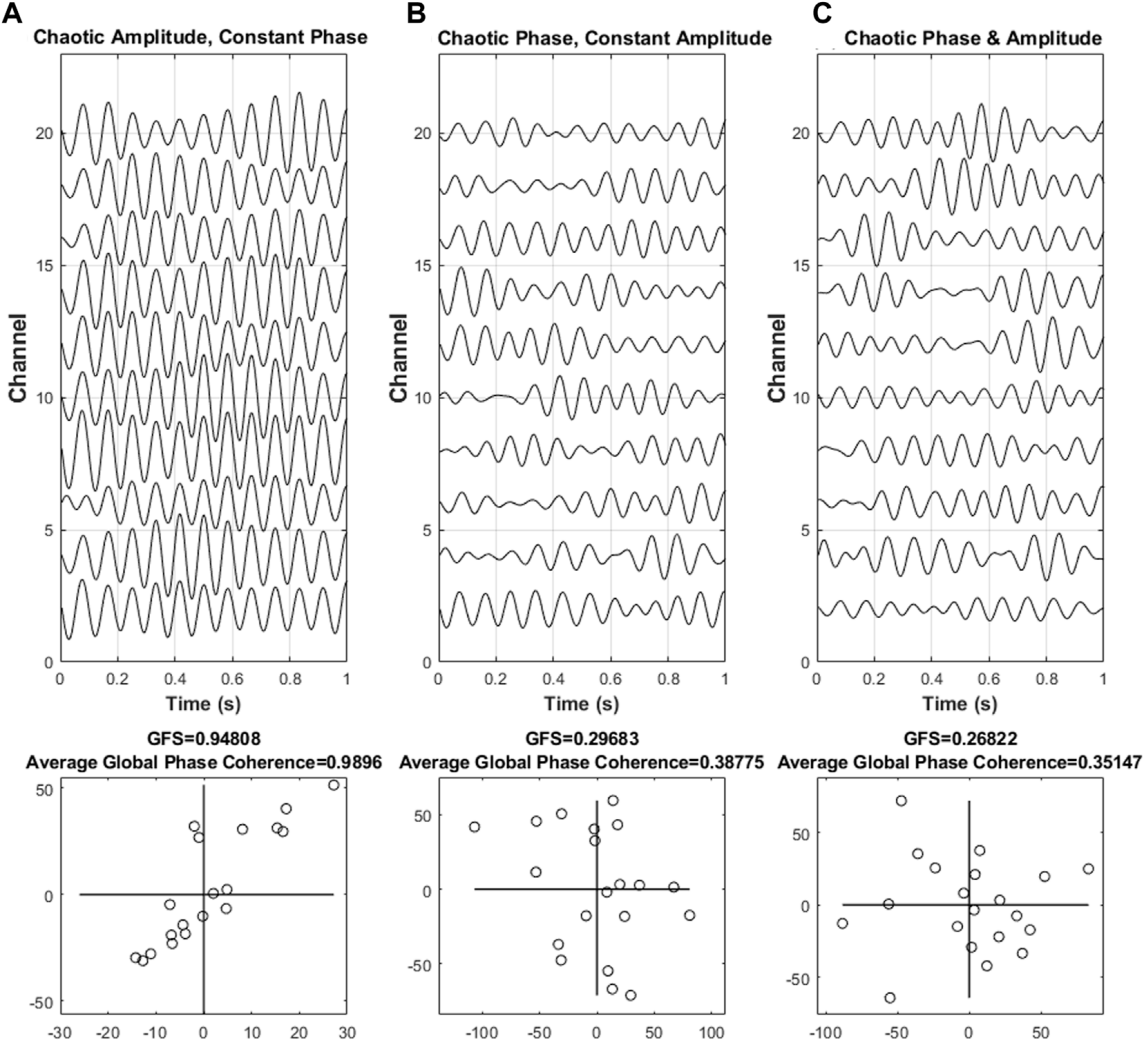
Different scenarios of synchronization. Three different scenarios of synchronization, such as **(A)** phase synchronization with chaotic amplitude, **(B)** constant amplitude with chaotic phase synchronization, and **(C)** chaotic amplitude and phase, with the corresponding complex plane that reflects the intensity are shown. The shape of the points in the diagram implies the intensity of the phase synchronization of the resulting values. An elongated form of the points indicates that the majority of the signal was dominated by a single phase angle at a specific frequency, thus indicating the presence of phase synchronization. On the other hand, the unsynchronized phase results in an almost round form.

In addition, we also used signals to validate that the GFS is inclined to be disrupted by phase, instead of amplitude. Based on the band-limited signal model (Eq. 1), we generated a 20-channel signal (only showing 10 channels in Figure 2). By manipulating the modulation function $\varphi_{i}\left(t\right)$ in the phase, we could generate a signal model with different scenarios of synchronization. Each signal goes through a band-pass filter after phase adjustment. Then, these signals could derive the GFS score. On the other hand, we calculated the phase coherence between each channel (Eq. 21) averaged the total phase coherence and then denoted it as global phase coherence so that we were able to compare the relationship between GFS and the global phase coherence toward amplitude and phase.

In this simulation, we proposed three situations to discuss, namely, (A) phase synchronization with chaotic amplitude, (B) constant amplitude with chaotic phase synchronization, and (C) chaotic amplitude and phase.

In Figure 2, the result indicated that as our previous assumption, the value of GFS is vulnerable to phase disturbance but not to amplitude disturbance. Furthermore, as the phase begins to become chaotic, both GFS and global phase coherence decreased. In simple words, the GFS, which may have a similar function as global phase coherence, is able to distinguish synchronization or not. In addition, both global phase coherence and GFS can evaluate the degree of synchronization.

### 3.2 Sensitivity Performance Comparison of GFS and Global Phase Coherence

As seen in the previous result, we have already discovered that the GFS is sensitive to phase distribution, and GFS somehow has a relationship with the phase coherence. Based on the process in Section 3.1.1, we also adapted a 20-channel signal model. In this section, we altered the strength of modulation function $\varphi_{i}\left(t\right)$ (Eq. 1) with the constant amplitude. We assumed $\varphi_{i}\left(t\right)$ as a random variable with normal distribution and used 10 different standard distributions ( σ : 0:2π ), that is, we were going to observe the score of GFS and global phase coherence during the phase manipulation.

**FIGURE 3:**
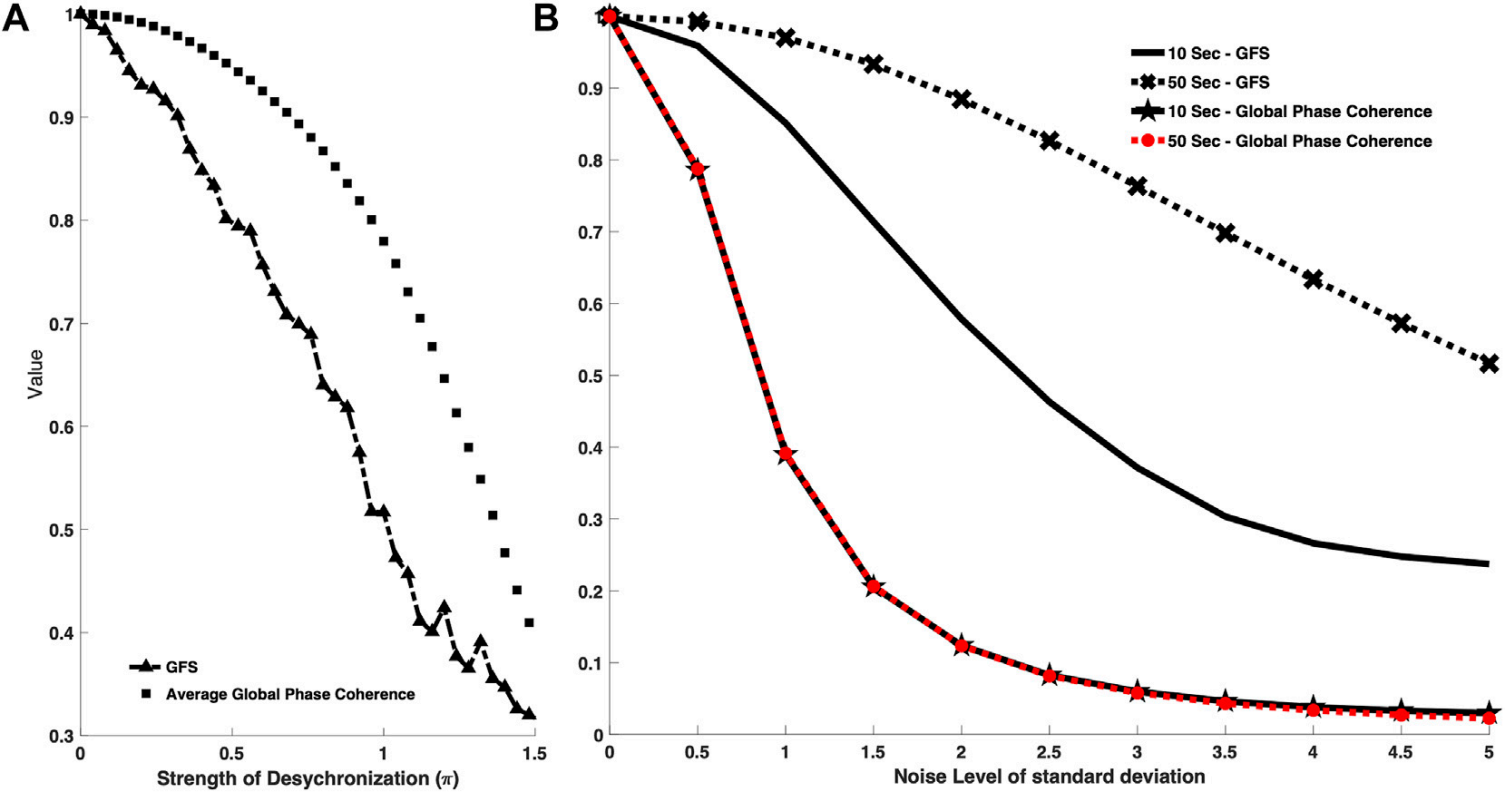
**(A)** Comparison between GFS and global phase coherence toward different strengths of frequency modulation function. The square-marker line shows the phase coherence curve, while the triangle-marker line represents the GFS curve. The *x* axis is the strength of frequency modulation value. The higher the *x* axis value, the less synchronized the signal model. GFS has a similar function with the phase coherence–based model and is able to recognize the degree of the modulation. **(B)** Comparison between GFS and global phase coherence toward different data lengths. The cross-marker line shows the GFS curve with a data length of 50 s, while the black line represents the GFS curve with a data length of 10 s. The star-marker line shows the global phase coherence curve with 10-s data length while the red-dotted line represents the global phase coherence curve with 50-s data length. The *x* axis is the noise level of standard deviation. The higher the *x* axis value, the higher the noise level. Both GFS and global phase coherence decayed as the noise level increased. GFS can have better robustness with longer data, while the data length cannot improve the performance of phase coherence.

The signals become chaotic when standard distribution of $\varphi_{i}\left(t\right)$ increase, so it could also be regarded as a desynchronization indicator. The result in Figure 3A revealed that both GFS and global phase coherence decrease when the strength of standard distribution of $\varphi_{i}\left(t\right)$ (desynchronization indicator) increases. Therefore, GFS has a similar function with the phase coherence–based method. Nonetheless, the curve of GFS decays faster than the curve of global phase coherence to the strength of desynchronization. On the other hand, GFS was proven as a much sensitive measurement tool than global phase coherence.

Moreover, in addition to the comparison in sensitivity of both methods to signal synchronization, we also performed a comparison of the sensitivity to the length of the signal. In clinical scenarios, EEG is vulnerable to different kinds of noise, which may badly affect the resulting GFS scores. Based on our proposed robustness algorithm, we could divide signals into several segments and reject the noise events, that is, the longer signals help us remove the noise events and acquire a genuine GFS score.

In addition, we not only considered the temporal noise event but also made GFS robust to the poor signal quality condition. We added different levels of noise into signals to study the robustness between different signal length and the background noise. (Figure 3B).

In Figure 3B, we tested 50-s and 10-s signals with the phase coherence–based method and GFS under different SNR scenarios. Though both methods decreased as the noise level increased, changes in signal length do not affect the global phase coherence score result, while longer signals showed significant improvement in the GFS score.

### 3.3 Baseline Characteristics of the Study Population

**FIGURE 4:**
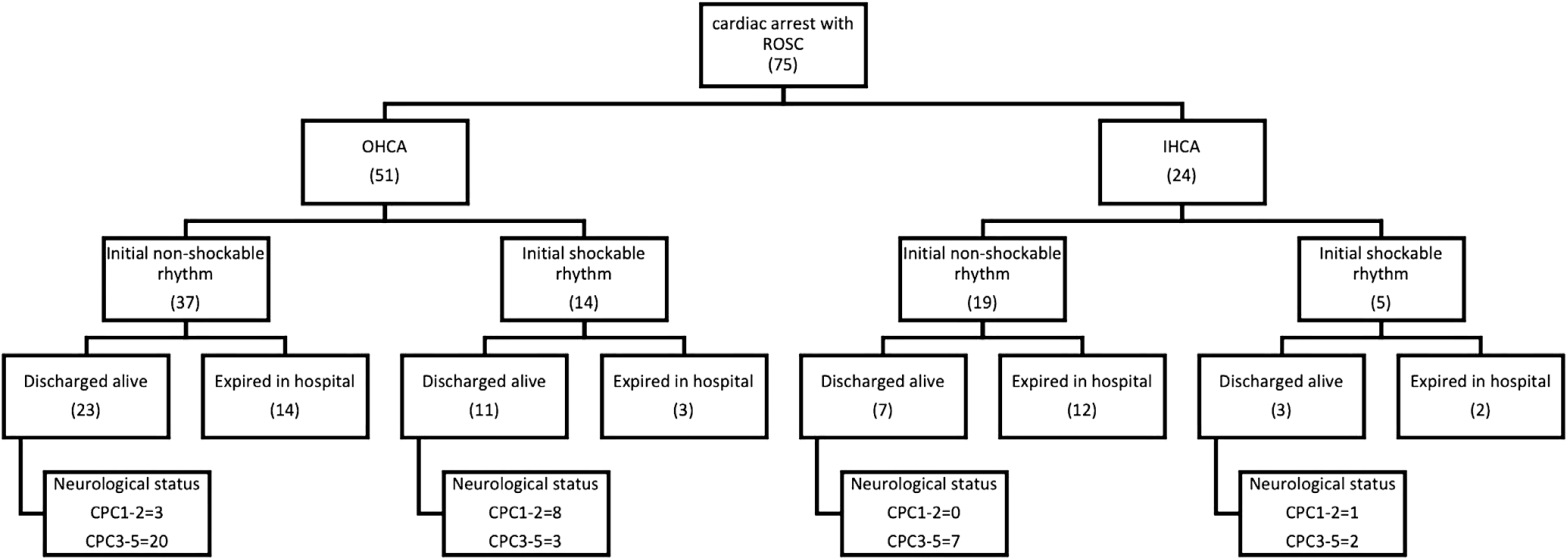
Patient demographics and clinical parameters.

The demographics and clinical parameters of all SCA survivors are shown in Table 1 and Figure 4. The mean age in the good-outcome group was younger than that in the poor-outcome group (51.6 ± 15.7 vs 68.1 ± 12.9, *p* < 0.001). The patients in both the groups were male-dominant. The patients with initial shockable rhythm were more in the good-outcome group (75 vs 15%, *p* < 0.001). The patients in the good-outcome group had a higher percentage of receiving TTM than that in the poor-outcome group (83.3 vs 44.4%, *p* = 0.014). The APACHE II score was higher in the poor-outcome group (25.4 ± 6.2 vs 19.3 ± 5, *p* = 0.0019). There was no statistical difference in the percentage of OHCA, duration of CPR, and rate of receiving ECMO therapy between the two groups. There was also no statistical difference in rates of comorbidities including CAD, DM, HTN, CKD, and AF between the two groups. Multivariate logistic regression analysis showed that only initial shockable rhythm was an independent factor for predicting good outcome (OR 2.15, 95% CI 1.6–46.1, *p* = 0.012).

**TABLE 1:** Patient demographics and clinical parameters.

Variable	Outcome		*p* Value
	Good outcome (n = 12)	Poor outcome (n = 63)	
**Age**	51.6 (15.7)	68.1 (12.9)	<0.001
**Year (SD)**			
**Male**	10 (83.3)	39 (61.9)	0.13
**N (%)**			
**OHCA**	11 (91.7)	40 (63.5)	0.049
**N (%)**			
**Shockable rhythm N (%)**	9 (75)	10 (15)	<0.001
**CPR duration mean (SD)**	18.8 (13.7)	18.7 (11.8)	0.98
**TTM**	10 (83.3)	28 (44.4)	0.014
**N (%)**			
**ECMO**	0 (0)	4 (6.3)	0.49
**N (%)**			
**APACHE II**	19.3 (5)	25.4 (6.2)	0.0019
**Mean (SD)**			
**CAD**	6 (50)	19 (30.2)	0.158
**N (%)**			
**DM**	1 (8.3)	26 (41.3)	0.026
**N (%)**			
**HTN**	7 (58.3)	30 (47.6)	0.358
**N (%)**			
**CKD**	0 (0)	15 (23.8)	0.054
**N (%)**			
**AF**	0 (0)	11 (17.5)	0.126
**N (%)**			

OHCA: out-hospital cardiac arrest; CPR: cardiac pulmonary resuscitation; TTM: target temperature management; ECMO: extracorporeal membrane oxygenation; APACHE II: Acute Physiology and Chronic Health Evaluation II; CAD: coronary artery disease; DM: diabetes mellitus; HTN: hypertension; CKD: chronic kidney disease; AF: atrial fibrillation.

### 3.4 Electroencephalogram Results

#### 3.4.1 Electroencephalogram as a Biomarker to Differentiate Good and Bad outcome—Qualitative Electroencephalogram

Qualitative EEG analyses showed that out of 12 patients in the good-outcome group, only two presented with benign EEG patterns, and the other 10 EEGs presented with a malignant pattern. All patients in the poor-outcome group had malignant or highly malignant pattern EEGs. The malignant and highly malignant pattern showed 100% sensitivity and 17% specificity to predict the poor outcome, respectively. The positive predictive value (PPV) was 0.86, and the negative predictive value (NPV) was 1. On the other hand, benign EEG showed 17% sensitivity and 100% specificity to predict good outcome. PPV was 1, and NPV was 0.86 (Table 3).

#### 3.4.2 Electroencephalogram as a Biomarker to Differentiate Good and Bad outcome—Quantitative Electroencephalogram

**FIGURE 5:**
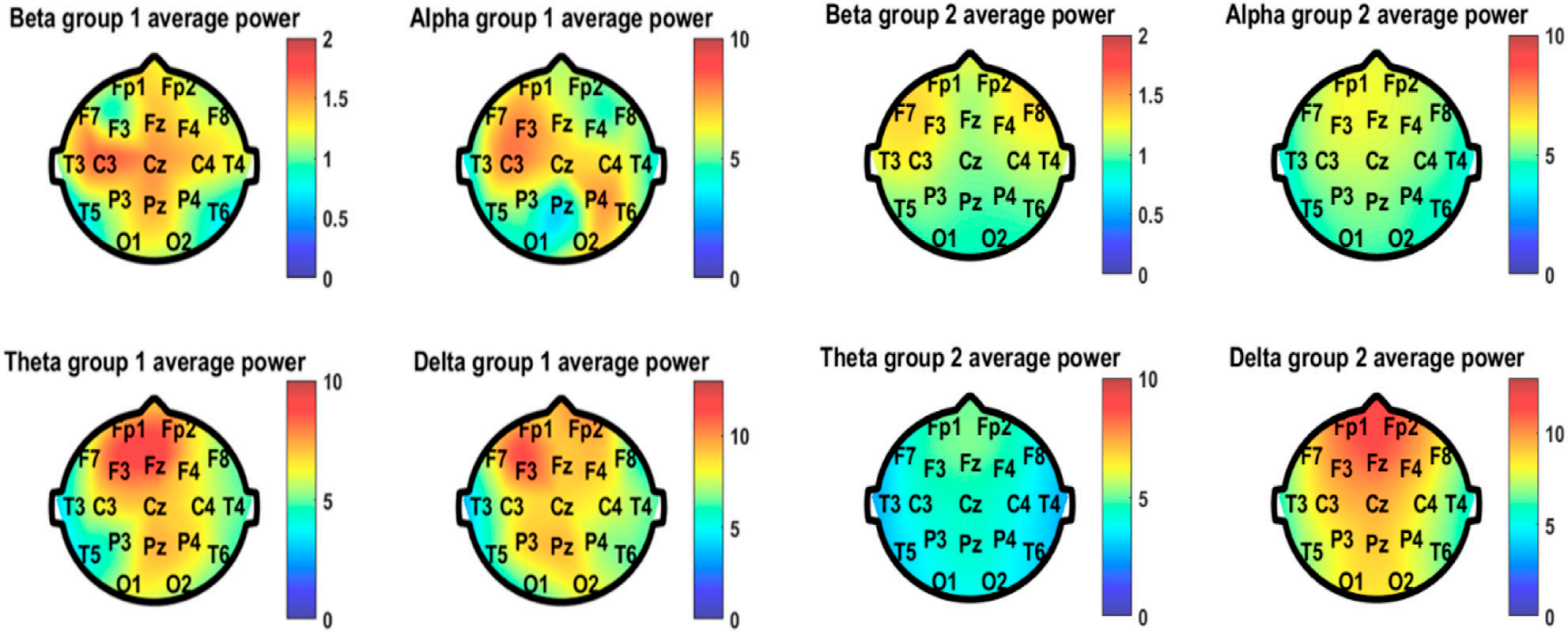
Average power of the four frequency bands of EEG spectral analysis in different positions of both group 1 (good outcome) and group 2 (bad outcome).

As for the quantitative EEG analyses, the average power of the four frequency bands of EEG spectral analysis in different positions of both groups is shown in Figure 5. The good-outcome group displayed higher power at each position in all four frequency bands. The values of average power of the four bands in the two groups are listed in Table 2.

**TABLE 2:** Values of average power of the four bands in the two outcome groups. G1 (Good Outcome) and G2 (Bad Outcome).

Frequency Bands **(**in **hz)**		Channel																		
		**1**	**2**	**3**	**4**	**5**	**6**	**7**	**8**	**9**	**10**	**11**	**12**	**13**	**14**	**16**	**17**	**18**	**19**	**20**
		**F4**	**C4**	**P4**	**O2**	**F3**	**C3**	**P3**	**O1**	**F8**	**T4**	**T6**	**F7**	**T3**	**T5**	**Fp1**	**Fp2**	Fz	Pz	Cz
**Delta (>4)**	G1	2.278	2.429	2.15	2.187	0.107	0.104	0.11	1,186	2.167	2.157	2.157	0.157	0.137	1.00E+05	0.136	9,451	0.114	0.211	0.105
	G2	8.813	7.766	11.1	8.845	4.37	5.497	12.2	4.96	8.921	9.829	8.505	3.54	5.256	7.474	3.762	8.721	5.827	6.501	5.373
	*p*-value	0.662	0.655	0.651	0.656	0.567	0.52	0.575	0.022	0.683	0.648	0.656	0.613	0.537	0.021	0.616	0.021	0.543	0.56	0.537
**Theta (4–8)**	G1	0.013	0.018	0.012	0.012	0.006	0.005	0.006	59.22	0.011	0.011	0.011	0.008	0.005	2,840	0.006	444.4	0.007	0.006	0.006
	G2	0.676	0.855	1.577	0.678	0.433	0.486	1.574	0.636	0.758	1.252	0.679	0.38	0.499	0.875	0.409	0.769	0.535	0.706	0.493
	*p*-value	0.516	0.546	0.606	0.537	0.525	0.511	0.586	0.023	0.596	0.603	0.564	0.6	0.541	0.021	0.596	0.021	0.533	0.564	0.525
**Alpha (8–12)**	G1	0.004	0.005	0.004	0.004	0.003	0.002	0.002	20.06	0.003	0.003	0.003	0.003	0.002	960	0.002	150.4	0.003	0.003	0.003
	G2	0.214	0.205	0.385	0.219	0.121	0.118	0.707	0.154	0.19	0.326	0.185	0.11	0.118	0.251	0.105	0.205	0.126	0.148	0.15
	*p*-value	0.554	0.535	0.592	0.543	0.532	0.488	0.613	0.022	0.593	0.606	0.566	0.592	0.534	0.021	0.571	0.021	0.495	0.548	0.543
**Beta (12–40)**	G1	0.004	0.004	0.004	0.004	6.00E-04	5.00E-04	4.00E-04	4.164	0.004	0.004	0.003	0.001	4.00E-04	199.3	6.00E-04	31.25	5.00E-04	6.00E-04	5.00E-04
	G2	0.035	0.031	0.067	0.034	0.024	0.024	0.109	0.029	0.031	0.055	0.033	0.019	0.026	0.046	0.021	0.039	0.026	0.028	0.026
	*p*-value	0.484	0.525	0.55	0.516	0.429	0.416	0.58	0.022	0.591	0.595	0.546	0.513	0.463	0.021	0.499	0.021	0.453	0.468	0.46

**FIGURE 6:**
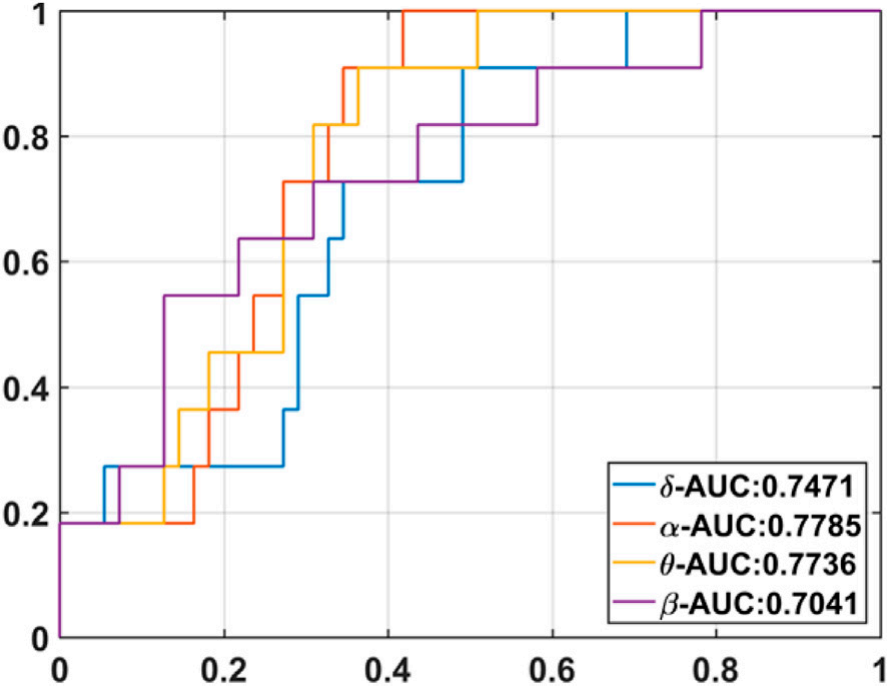
Area under curve (AUC) in different frequency bands.

The area under the curve (AUC) value for power in different frequency bands was also calculated (Figure 6). Among the four frequency bands, the alpha band showed the highest discrimination ability (AUC = 0.78) to predict good outcome. The sensitivity of EEG power was 100%, specificity was 58%, PPV was 0.32, and NPV was 1 to predict good outcome (Table 3).

**TABLE 3:** Sensitivity, specificity, PPV, and NPV values of each parameters to predict good outcome.

	Sensitivity	Specificity	PPV	NPV
**Qualitative analysis (Benign pattern)**	0.17	1	1	0.86
**Power**	1	0.58	0.32	1
**GFS**	0.90	0.60	0.31	0.97
**Combined power + GFS**	0.73	0.93	0.67	0.94

PPV: positive predictive value; NPV: negative predictive value; GFS: global field synchronization.

#### 3.4.3 Correlation of Global Field Synchronization to Sudden Cardiac Death Survivor’s Outcome

**FIGURE 7:**
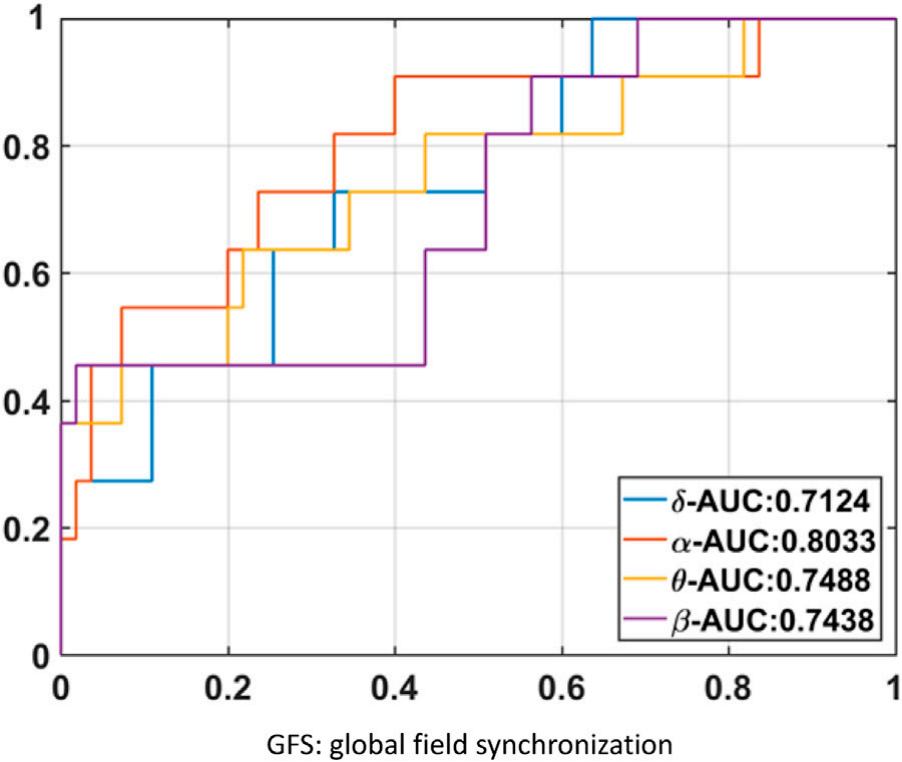
GFS AUC value for each frequency band.

The results of GFS analysis are listed in Table 4. As shown in Table 4, the GFS values were significantly higher in the good-outcome group in all four frequency bands. The AUC value for each frequency band is shown in Figure 7.

**TABLE 4:** GFS values of two outcome groups.

Frequency Bands (Hz)	Neurological outcome		*p* Value
	Good outcome (n = 12)	Poor outcome (n = 63)	
**Delta (<4)**	0.4647	0.3490	<0.001
**Theta (4–8)**	0.4141	0.3302	<0.001
**Alpha (8–12)**	0.3956	0.3216	<0.001
**Beta (12–24)**	0.4015	0.3324	<0.001

GFS: global field synchronization.

**FIGURE 8:**
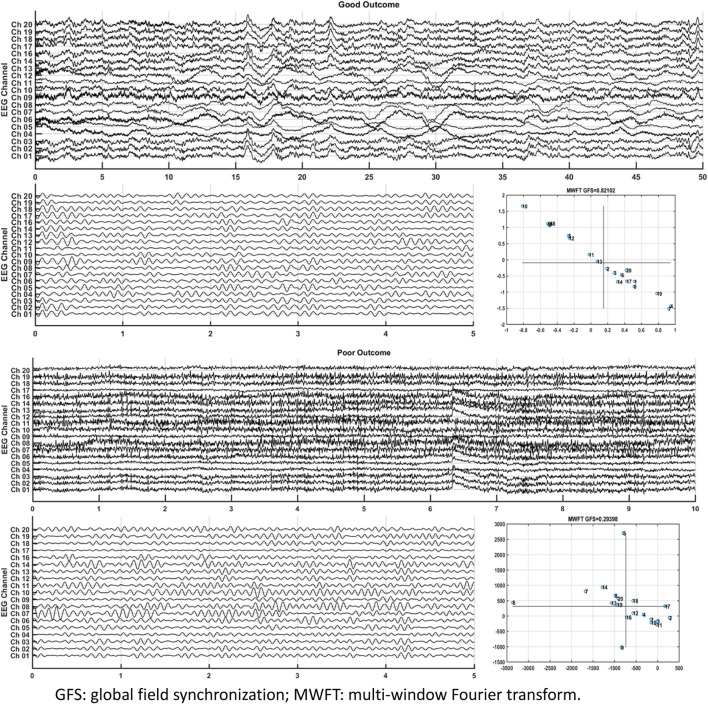
Examples of real data for both groups.

GFS of the alpha band showed the highest AUC value (0.8) to predict good outcome. Figure 8 shows examples of real data for both groups. Real data from the good-outcome group exhibit good phase synchronization, in spite of the randomness of its amplitude, which results in a high GFS value. This synchronization can be clearly observed in the alpha band plot, while for the poor outcome, the phase and amplitude synchronization observed was poor. The randomness of both the phase and amplitude contributes to the poor GFS result. The sensitivity of GFS was 90%, specificity was 60%, PPV was 0.31, and NPV was 0.97 (Table 3).

**FIGURE 9:**
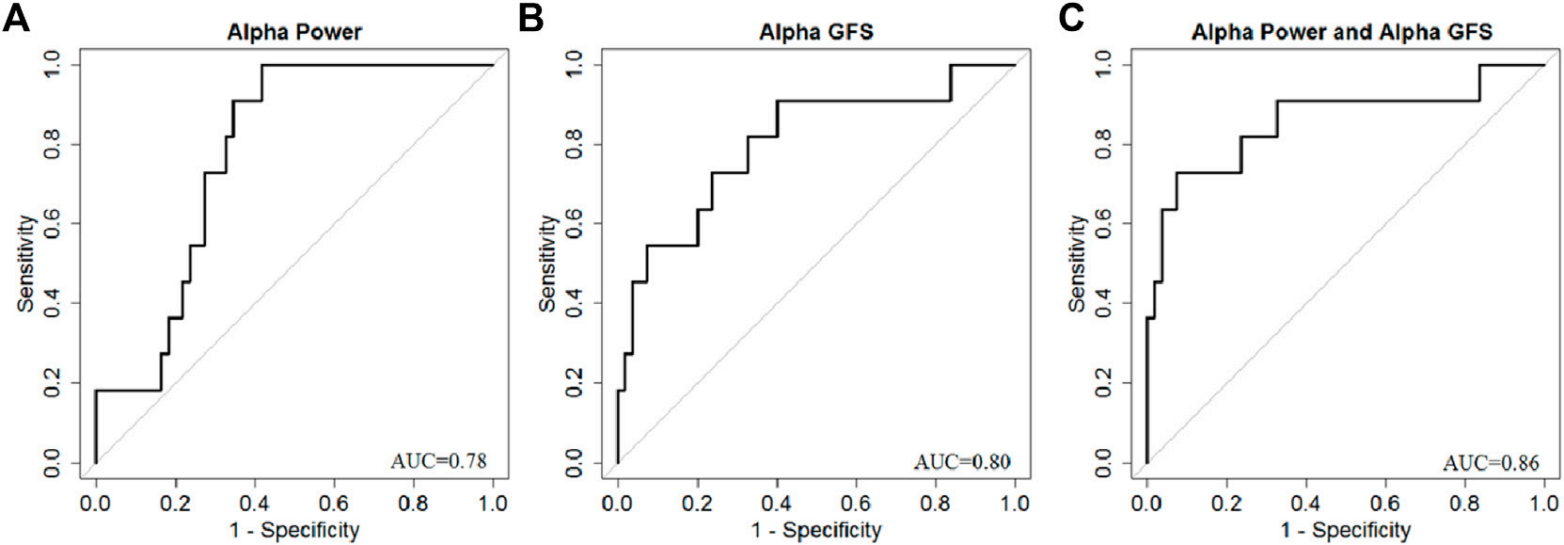
AUC of **(A)** Alpha band power, **(B)** Alpha GFS, and **(C)** Combination of EEG power and GFS in Alpha band as outcome predictors.

By combining EEG power and GFS, the alpha band power + GFS showed the best prediction value (AUC 0.86) in predicting good outcome (Figure 9). The sensitivity of EEG power + GFS was 73%, and specificity was 93% (Table 3).

## 4 Discussion

In this study, we presented from a mathematical point of view of why phase coherence plays an important part in GFS. Furthermore, data simulation supports the mathematical proof we provided. In addition, we looked into qEEG measures and GFS as potential biomarkers to use to differentiate neurological outcome predictions in sudden cardiac death survivors. The results show the possibility of both measures in predicting the outcome significantly, whether good or bad.

### 4.1 Outcome Prediction in Comatose Patients Following Cardiac Arrest

In one previous study by Grmec and Gasparovic (2001), among the scoring systems including Mainz Emergency Evaluation System (MEES), Glasgow Coma Scale (GCS), and APACHE II, GCS performed the best in the prediction of mortality for nontraumatic coma patients. Schefold et al. (2009) also considered GCS as a powerful tool in predicting the neurological outcome of cardiac arrest patients treated with therapeutic hypothermia. APACHE II (Acute Physiology and Chronic Health Evaluation II) is a severity-of-disease classification system that includes a GCS score as part of evaluation. The APACHE II, although it encompasses the GCS score as part of the evaluation, was only a modest indicator of illness severity and predictor of mortality/neurologic morbidity (Donnino et al., 2013). In this study, the APACHE II score was higher in the poor-outcome group in the univariate analysis but became insignificant after the adjustment of other covariates in regression analysis.

In this study, the mean age in the good-outcome group was significantly less. The patients in the good-outcome group had more initial shockable rhythm, higher percentage of receiving TTM, and lower APACHE II score. The previous studies showed that TTM increased the rate of a favorable neurologic outcome and reduced mortality (Lascarrou et al., 2019). However, the APACHE II score of patients with poor outcomes in our study was higher than that of patients with good outcomes. Therefore, more patients in the poor-outcome group might have contraindications of TTM, and the multivariate logistic regression analysis showed that only initial shockable rhythm was an independent factor for predicting good outcome, rather than TTM or not or APACHE II score.

The prediction of clinical outcomes will help optimize the treatment and benefit the patients. In the clinical setting, neurocritical illnesses are diseases requiring expensive treatment associated with poor outcomes. Survival with poor neurological outcomes is very resource-demanding. The treatment plans may be altered by the socioeconomic condition of the patient or his family. The family and clinician may avoid futile medical care if poor outcomes can be identified early. On the other hand, if we can recognize patients who are potential independent survivors, aggressive treatment is indicated, and the patients will be beneficial to the treatment.

### 4.2 Limitation of Qualitative Electroencephalogram in Neurological Outcome Prediction

Neurologists interpret EEG according to the ACNS EEG terminology (Westhall et al., 2016) as qualitative EEGs. In this study, highly malignant and malignant patterns both showed high sensitivity but low specificity in predicting poor outcome. Continuous background indicates good outcome, which is defined as continuous normal voltage (>20 μV) background and preserved reactivity to stimuli. In this study, benign patterns showed high specificity but low sensitivity. There is interrater variability in qualitative EEG interpretation. Westhall et al. (2015) published a multinational study on the interrater reliability of EEG interpretation using the 2013 revised ACNS terminology among comatose patients following cardiac arrest. There was a high interrater agreement (κ 0.71) for highly malignant patterns and moderate agreement (κ 0.42) for malignant patterns. Identifying an unreactive EEG was fair (κ 0.26). Therefore, traditional qualitative evaluation of EEG should be interpreted with caution. Using quantitative methods may lower the interrater variation. Duez et al. (2018) published a quantitative method to evaluate EEG reactivity. In the study, agreement among experts on overall EEG reactivity varied from 53 to 83% (κ 0.05–0.64) and reached 100% (κ1) between two quantitative EEG reactivity calculators.

### 4.3 Relationship of Quantitative Electroencephalogram to the Variability of Neural Activities in Discrimination Between Good and Bad Prognosis

Neurophysiological monitoring tests, such as EEG and somatosensory evoked responses (SSEPs), may play a role in the prediction of neurological outcome prediction (Rossetti et al., 2016). EEG is a signal with a complex structure, and several EEG features have been proven to be correlated with the degree of neuronal injury (Westhall, 2017). However, EEG signal evaluation requires specific expertise and can suffer from interrater variability (Westhall et al., 2015). Computational processing of the EEG or quantitative EEG (qEEG) has been utilized for neurological outcome prediction after cardiac arrest. The parameters derived from qEEG such as bispectral index (BIS), amplitude-integrated EEG, burst-suppression ratio, or entropy, have been demonstrated to be valuable in clinical practice (Leary et al., 2010; Rundgren et al., 2010; Seder et al., 2010; Noirhomme et al., 2014; Selig et al., 2014; Stammet et al., 2014). Among the parameters, some studies showed that BIS had better discriminative power in predicting the outcome (Leary et al., 2010; Seder et al., 2010; Selig et al., 2014; Stammet et al., 2014), while other computer-assisted analyses had high specificity but relatively low sensitivity for outcome prediction.

In this study, we demonstrated that patients with a good outcome had higher EEG power in all four frequency bands. The higher EEG power represents higher variability of neural activities and might be associated with better brain function after cardiac arrest. Our result also showed that among the frequency bands, the power of the alpha band was the best in predicting the outcome. This result was correlated with visual analysis findings (Westhall, 2017) and was compatible with previous studies (Wiley et al., 2018; Kota et al., 2020).

Alpha waves are neural oscillations in the frequency range of 8–12 Hz, likely originating from the synchronous and coherent electrical activity of thalamic pacemaker cells in humans (Hughes and Crunelli, 2005). Alpha waves appear in the EEG during the resting but wakeful state, while beta waves are seen in more alert status, and delta/theta waves are seen in the sleep state. In this study, the alpha band showed the best predictive value among all four frequency bands, which is compatible with the clinical neurological status of these patients: comatose but going to wake.

### 4.4 Significance of Phase Coherence to Global Field Synchronization

Understanding the mathematical perspective of a system describes how a system works. It uncovers the underlying essential aspects. Based on the original concept by Koenig et al. (2001), GFS focuses on the local distribution between phase and amplitude variation. They explained that channels are synchronized if they have the same phase, counter phase, and a much elongated distribution on the sine–cosine plane, which could indicate a higher degree of synchronization. The data simulations presented in this study have proven the initial claim that both parameters, indeed, play a part in the calculation of GFS. The distribution of amplitude may determine the tolerance of phase variation with the GFS method. Wide amplitude variation signals could get a higher GFS score with wider phase variation than the narrow amplitude variation signals. However, regardless of the amplitude variation, the phase distribution is still the dominant factor to decide the GFS score. Figure 1 shows that the narrower the distribution for phase, the higher the value for GFS, while for amplitude, an increase in amplitude does not correlate to higher GFS if the distribution of phase is wide. This claim is true to both traditional GFS and GFS without the zero-mean process. Hence, although both amplitude and phase contribute to obtaining GFS, interference in phase variation drastically changes the possibility of generating a good GFS score.

In addition, we want to address the critical limitation of the GFS method. When the different channels in a signal share uniform amplitude, even though they have a perfect phase-locking effect, the resulting GFS score would decrease. At the same time, GFS will not be able to detect a perfect phase-locking signal with uniform phase and signals with random phase and amplitude. Fortunately, in clinical applications, we usually record multichannel EEG signals, and it is rare for the amplitude of the different electrodes to have equal magnitude.

Furthermore, in Figure 2, we showed different scenarios of simulated data and provided the sine–cosine plot for GFS. Moreover, the values for GFS and phase coherence were also included. Comparing all scenarios, GFS and phase coherence were at their highest at constant phase even though the amplitude varies for every channel. The random phase with constant amplitude resulted in a much lower value both in GFS and phase coherence compared to data simulation with constant phase due to more random phases related to wider distribution. In addition, we showed that the two measures declined as the interference gets more chaotic, but GFS showed more sensitivity than the phase coherence measure in terms of increased interference. Moreover, longer signal length has better robustness to attenuate the background noise affect in the GFS method, resulting in a better GFS score since we could also reject the spike-like artifact in longer length, though this scenario does not apply with global phase coherence.

### 4.5 Neurological Outcome Predictions With Global Field Synchronization

GFS is a novel method to analyze the functional synchronization of brain activity recorded by EEG, which was introduced by Koenig et al. (2001). GFS can estimate the connectivity of brain electric activities among different brain regions and is effective in the diagnosis of some neurologic or psychiatric diseases such as Alzheimer’s disease (Ma et al., 2014) or obsessive–compulsive disorder (Ozcoban et al., 2018). To our knowledge, our study is the first one to utilize GFS as an EEG analysis method for neurological outcome prediction in cardiac arrest survivors. In addition, we believe that we are the first to present and explain the true dynamics between GFS and phase coherence from a mathematical point of view. Delving deeper into the method gives new perspective and an in-depth understanding on how it works. Based on the mathematical review presented in the *Materials and Methods* section of this article, we provided solid proof that GFS is mainly based on phase coherence than its amplitude. Furthermore, based on the results, among the four frequency bands, the alpha band showed the best sensitivity and specificity to predict neurological outcome. By combining EEG power and GFS, the predictive value is even better. This implies that the connectivity of brain electric activities is an important marker of brain function after cardiac arrest.

## 5 Conclusion

There were some limitations to this study. First, this was a retrospective study with a small study population. However, according to the power analysis, the size of population was enough for the evaluation of the diagnostic ability. Second, the timing of performing EEG was not uniform in all patients. Late or early EEG might reveal difference results. Third, the EEG in this study was a standard EEG recording for 5 min rather than continuous EEG, which represented the brain activity only at that period of time without a dynamic change.

In conclusion, this is the first study to use GFS to predict neurological outcome in cardiac arrest survivors and present solid proof that GFS is, indeed, based on phase coherence. By combining GFS and EEG power analysis, the neurological outcome of the nontraumatic cardiac arrest survivor can be well-predicted.

## Data Availability

The raw data supporting the conclusions of this article will be made available by the authors, without undue reservation.
